# Blind Fractionally Spaced Channel Equalization for Shallow Water PPM Digital Communications Links

**DOI:** 10.3390/s19214604

**Published:** 2019-10-23

**Authors:** Gaetano Scarano, Andrea Petroni, Mauro Biagi, Roberto Cusani

**Affiliations:** DIET, Università “La Sapienza” di Roma, via Eudossiana 18, 00184 Roma, Italy; gaetano.scarano@uniroma1.it (G.S.); mauro.biagi@uniroma1.it (M.B.); roberto.cusani@uniroma1.it (R.C.)

**Keywords:** fractional sampling, channel equalization, Bussgang, PPM

## Abstract

Underwater acoustic digital communications suffer from inter-symbol interference deriving from signal distortions caused by the channel propagation. Facing such kind of impairment becomes particularly challenging when dealing with shallow water scenarios characterized by short channel coherence time and large delay spread caused by time-varying multipath effects. Channel equalization operated on the received signal represents a crucial issue in order to mitigate the effect of inter-symbol interference and improve the link reliability. In this direction, this contribution presents a preliminary performance analysis of acoustic digital links adopting pulse position modulation in severe multipath scenarios. First, we show how the spectral redundancy offered by pulse position modulated signals can be fruitfully exploited when using fractional sampling at the receiver side, which is an interesting approach rarely addressed by the current literature. In this context, a novel blind equalization scheme is devised. Specifically, the equalizer is blindly designed according to a suitably modified Bussgang scheme in which the zero-memory nonlinearity is replaced by a *M*-memory nonlinearity, *M* being the pulse position modulation order. Numerical results not only confirm the feasibility of the technique described here, but also assess the quality of its performance. An extension to a very interesting complex case is also provided.

## 1. Introduction

Intersymbol interference (ISI) represents the main impairment in wireless data links over multipath channels. In the specific context of underwater acoustic communications (UWAC), attenuation, noise and multipath affecting the sound propagation cause a large delay spread, severely degrading the quality of the received signal [1]. Therefore, ISI mitigation is crucial in order to provide good communication performance. In this direction, digital filtering at the receiver side aims at achieving the so-called channel equalization condition, so that the received signal is reshaped as close as possible to the transmitted one. Due to the distortion disturbing the received signal, channel equalization becomes necessary in underwater acoustic communications. This is true especially when dealing with shallow water scenarios where the multipath trajectories exhibit short path lengths before reflections, thus making ISI cancellation very challenging.

Regarding such points, the literature presents several solutions, many of which are based on the implementation of decision feedback equalizers (DFEs). This kind of filtering, belonging to the category of nonlinear equalizers [2], is used when the signal distortion caused by the channel can not be reliably mitigated by linear equalizers. The DFE structure is based on a feed-forward filter (FFF) cascaded with a feedback filter (FBF), and allows not only the cancellation of ISI on the current received symbol, but also the estimation of ISI caused by the current symbol on the future ones, so that ISI can be subtracted out before the next symbol detection. The fast convergence provided by DFEs is paid in terms of computational complexity since the coefficients updating concerns two filters instead of a single one, as in the case of linear equalization. Moreover, complexity depends also on the adaptation rule used for filter updating. Consider in fact that the least mean square (LMS) algorithm, the simplest to realize, requests a computational cost proportional to 2N, while the recursive least squares (RLS) algorithm, is faster than LMS, and the computational complexity is proportional to 20N, N being the total number of equalizer coefficients. An example of DFE applied to single-carrier UWAC is reported in [3], where an iterative frequency domain equalization combined with low density parity check (LDPC) decoding is presented. Numerical results show that the use of coding speeds up the equalizer convergence, while also providing 6 dB final gain. Moreover, the combined equalization and decoding processing allows the achievement of better performance than the detection scheme where equalization and decoding are performed sequentially.

In general, the stronger the ISI, the higher the equalizer requested performance and, therefore, the higher the receiver complexity. This is an important aspect to take care of since the cost of hardware makes devices employed in underwater applications quite expensive, so the pursuit of a good trade-off between performance and complexity is fundamental.

A possible way to reduce the processing effort at the receiver side is to choose a suitable transmission scheme that considers the introduction of a guard interval between consecutive symbols. By doing so, the delay spread on the current symbol has enough time to expire, possibly limiting the superimposition with the next transmitted symbol, and thus reducing the ISI. On the other hand, as the presence of large recovery times puts the transmission in stand-by for most of the time, the data-rate is unavoidably reduced. Furthermore, although this solution may be reasonable in RF-based technology, it becomes instead harmful when dealing with UWAC, in this case being the date-rate already penalized by the speed of sound five orders of magnitude lower than the speed of light. While in single-carrier based communications the use of guard intervals can be conveniently replaced by adopting other techniques like channel coding [4], it is essential in multi-carrier based ones. However, this fact is very often underestimated, leading to erroneously recognize orthogonal frequency division multiplexing (OFDM) as definitively outperforming single-carrier schemes without any proper contextualization. In fact, the effectiveness of any transmission technique should be evaluated by properly taking into account the application scenario. We therefore deem it necessary to highlight some aspects of OFDM and single-carrier based communications.

### 1.1. Potential Limits of OFDM

OFDM is recognized as one of the most effective transmission schemes to achieve high data rates in UWAC [5,6,7]. However, as the characteristics of the underwater acoustic channel differ from scenario to scenario (in fact, there is no univocal and stable model for channel characterization [1,8]), OFDM is not expected to always achieve a satisfactory performance. In detail, in order to counterbalance the effect of ISI, OFDM symbol transmission is preceded by a cyclic prefix (CP), the length of which approximates the channel delay spread. Typically, the CP takes about 1/3 of the overall symbol length. But when referring to shallow water scenarios, it may happen that the channel delay spread is even larger than hundred of milliseconds, making the CP length occupying the largest part of the entire OFDM symbol time, and therefore reducing the transmission rate. Moreover, if the OFDM symbol length is larger than the channel coherence time, OFDM symbol synchronization may be lost and/or channel estimation may be not sufficiently accurate. Consequently, OFDM is combined with additional techniques in order to improve the communication reliability, achieved at the expense of a receiver complexity increase. An example is discussed in [9], where the considered iterative signal detection and decoding is performed by means of Doppler compensation, soft minimum mean square error (MMSE) equalization and LDPC channel decoding. The work in [10] addresses instead, another challenging issue for OFDM, that is phase synchronization combined with channel estimation. For the reasons highlighted above, OFDM based communications seem not to provide a convenient trade-off between rate, reliability, and complexity when dealing with strong, time-variable multipath channels.

### 1.2. Merits of Single-Carrier Schemes

The implementation of any single-carrier based transmission requires lower complexity than OFDM based schemes. In particular, pulse position modulation (PPM) is known to be particularly robust to ISI thanks to its time-frequency properties, allowing the achievement of good performance in terms of channel equalization without the necessity of using CP and/or guard intervals between symbols. In this direction, DFE in PPM based communications, even though not specifically related to the underwater scenario, is introduced in [11], where a chip-oriented *M*PPM transmission is considered, *M* being the number of chips per symbol. Since *M*PPM signals can be described as realization of cyclostationary random processes, in [11] the implementation of a bank of *M* DFEs is also suggested, each one working on a single chip of the *M*PPM symbol and characterized by a different set of coefficients. However, despite the fact that each DFE works only once every *M* chips, the receiver architecture remains quite complex. It is worth noting that most of works reported in the literature deal with filtering operated at symbol time—chip time in the case of the PPM scheme. This choice leads to a not-negligible and problematic time sensitivity that affects the overall system performance.

A possible way to overcome this problem is sampling the received signal at rates greater than the nominal one before filtering. This technique is known as fractionally spaced channel equalization [12], and the resulting filter is the so-called fractionally spaced equalizer (FSE) [13]. A direct application of this concept in underwater contexts is found in [14], where it is described as an improved version of the RLS constant modulus algorithm (CMA) ruling an FSE. In particular, a modified cost function of the CMA is introduced, which results in both faster convergence and computational complexity reduction with respect to the standard RLS-CMA. In addition to ISI cancellation, fractionally spaced equalization allows a more efficient symbol timing recovery, see [15,16] for more details. The work in [15] presented a method for symbol timing recovery by tracking the shift of the equalizer taps. With the scheme described in [16], synchronization is restored by estimating the time derivative of the equalizer output modulus and then adjusting the timing offset in feedback mode through interpolation. However, equalization performance strictly depends on the initial phase the received fractionally sampled signal is locked to; to this regard, a blind sampling phase estimation technique has been presented in [17].

Stemming from the above reported remarks, this contribution addresses the problem of ISI cancellation in shallow water acoustic communications. To this aim, we present a preliminary feasibility study about PPM transmission combined with a novel FSE scheme that provides good performance in severe multipath channel scenarios where the use of OFDM appears nonviable. Firstly, we analyze the structure of *M*PPM signals and their peculiar spectral redundancy further exploited in a probabilistic fashion. Specifically, stemming from the “memory” possessed by PPM signals, we devise a novel blind adaptive, modified Bussgang, FSE scheme, i.e., not data aided, whose feasibility and accuracy is assessed by numerical simulations. In detail, the modified Bussgang approach considers the introduction of an *M*-memory nonlinearity, never addressed before, in place of the classical zero-memory nonlinearity. Moreover, since another relevant issue in fractional sampling is the choice of the sampling phase, we resort to the mechanism described in [17] as part of the equalizer architecture. We remark that all the here presented matter are neither investigated nor mentioned in [17], which describes a sampling phase estimation technique here merely recalled, but not discussed or deepened at all. It is worth remarking that the use of PPM signals allows the described FSE scheme to be implemented in a blind fashion, while in OFDM based schemes certain known (pilot) symbols are exclusively used for channel estimation. Finally, we discuss the performance of different blind adaptive equalization schemes in terms of the speed of convergence and/or misconvergence percentage.

The paper is organized as follows: In Section 2 the structure of *M*PPM signals and their spectral characteristics are analyzed; in Section 3 the receiver operating at fractional sampling is presented, and in Section 4 the blind equalization scheme is described. Numerical results are presented in Section 5 and conclusions are drawn in Section 6. Finally, some analytical details are given in Appendices Appendix A and Appendix B.

## 2. *M*PPM Waveform

Loosely speaking, the peculiarity of *M*PPM signals is that each symbol is formed by *M* empty consecutive chips and one that is filled with a pulse. The position of such a filled chip encodes the transmitted information symbol.

In order to gain a more deep insight about the spectral structure of PPM signals induced by this peculiarity, we will show how *M*PPM signals can be expressed as particular pulse amplitude modulated (PAM) signals modulating a sequence of suitably correlated binary samples.

To this purpose, let us consider the *n*-th string $s_{n}\;=\;\left(b_{0,n},b_{1,n},\cdots ,b_{\nu -1,n}\right)$ collecting the $\nu \;=\;\log_{2}M$ bits to be transmitted after a suitable mapping to the corresponding *M*PPM symbol. This mapping is operated as follows: (*i*) Denoting by $j_{n}$ the decimal value of the binary number $s_{n}$, the $j_{n}$-th row of the identity *M*-matrix $I_{M}$ furnishes a *M*-tuple of binary valued *chips* $\left(c_{n}\left[0\right],c_{n}\left[1\right],\cdots ,c_{n}\left[M\;-\;1\right]\right)$; (*ii*) the *n*-th *M*PPM discrete symbol is formed as follows:

(1) $$c_{M\mathrm{PPM}}\left[n\right]\;=\;\sum_{m=0}^{M-1}c_{n}\left[m\right]\;\delta \left[n\;-\;m\right],\;n=0,1,\cdots ,M\;-\;1.$$

The *M*PPM discrete symbol expression (1) properly takes into account the fully-correlation existing between the *M* chip samples $\left(c_{n}\left[0\right],c_{n}\left[1\right],\cdots ,c_{n}\left[M\;-\;1\right]\right)$, M − 1 of which are 0 and the remaining equals 1.

Using (1), we can form a binary stochastic sequence that accounts for all the discrete symbols of a *M*PPM signal:

(2) $$b_{M\mathrm{PPM}}\left[n\right]=\sum_{k=-\infty }^{+\infty }c_{M\mathrm{PPM}}\left[n-kM\right]$$

For equiprobable bits, the direct component (DC) and the power of the sequence $b_{M\mathrm{PPM}}\left[n\right]$ respectively take the following values:

$$\left(\mathrm{DC}\right)\;M_{\;M\mathrm{PPM}}\;=\;\frac{1}{M}\;;\;\left(\mathrm{Power}\right)\;P_{\;M\mathrm{PPM}}\;=\;\frac{1}{M}.$$

The analog *M*PPM signal is then formed by interpolating the binary sequence $b_{M\mathrm{PPM}}\left[n\right]$ with a shaping pulse $g_{{}_{T}}\left(t\right)$ whose duration equals the chip-time $T_{c}$:

(3) $$s_{M\mathrm{PPM}}\left(t\right)=\sum_{n=-\infty }^{+\infty }b_{M\mathrm{PPM}}\left[n\right]\;g_{{}_{T}}\left(t-nT_{c}\right).$$

Since each *M*PPM symbol is formed every *M* consecutive chips, the symbol transmission rate is $F_{s}\;=\;1/MT_{c}$. More in detail, the form (3) states that analog *M*PPM signals can be expressed in terms of very particular binary PAM signals, which have the following, well known, power spectral density (PSD) [18,19]:

(4) $$P_{s_{M\mathrm{PPM}}}\left(j\Omega \right)=\frac{1}{T_{c}}{\left|G_{T}\left(j\Omega T_{c}\right)\right|}^{2}\;P_{b_{M\mathrm{PPM}}}\left(e^{j\Omega T_{c}}\right).$$

In (4), Ω = 2πf is the radian frequency, $G_{T}\left(j\Omega \right)$ is the Fourier transform of $g_{{}_{T}}\left(t\right)$, and $P_{b_{M\mathrm{PPM}}}\left(e^{j\Omega T_{c}}\right)$ is the PSD of the discrete binary random sequence of the *M*PPM symbols $b_{M\mathrm{PPM}}\left[n\right]$. To evaluate $P_{b_{M\mathrm{PPM}}}\left(e^{j\Omega T_{c}}\right)$, one can resort to the guidelines indicated in [18], or the more simple analytical technique developed in [19]:

(5) $$\begin{array}{cc} P_{b_{M\mathrm{PPM}}}\left(e^{j\Omega T_{c}}\right) & =\frac{1}{M}\left[1-{\left|\frac{\sin(\Omega T_{c}M/2)}{M\sin(\Omega T_{c}/2)}\right|}^{2}\right]+\frac{2\pi }{T_{c}\;M^{2}}\sum_{k=-\infty }^{+\infty }\delta \left(\Omega -2\pi k/T_{c}\right). \end{array}$$

**Figure 1:**
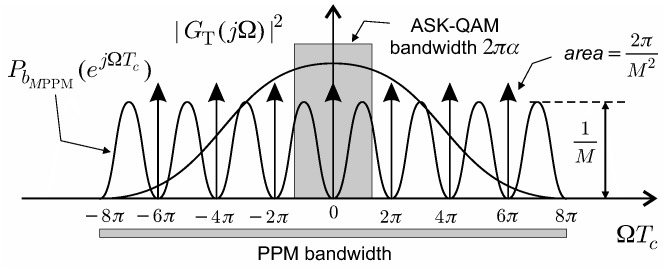
Power spectral density of *M*PPM signals. *M* position modulation (PPM) transmission, where *M* is the number of chips per symbol.

Figure 1 shows the rich spectral redundancy possessed by the PSD (4) of *M*PPM signals.

Generally speaking, due to the large bandwidth of the pulse $g_{{}_{T}}\left(t\right)$, the *M*PPM signal presents a sort of redundancy that consists in a special kind of spectral repetition coding. To highlight the *M*PPM signal redundancy, in Figure 1, we have also indicated the bandwidth occupied by a typical square root raised cosine (RRC) pulse employed in amplitude shift keying (ASK) or quadrature amplitude modulation (QAM) techniques. Interestingly enough, we observe that the spectral repetition coding realized by ASK-QAM only appears in the very small frequency band determined by the roll-off factor α of the RRC pulse—precisely of width $\alpha /T_{c}$ (Hz) around half the symbol rate $1/2T_{c}$. Moreover, since $b_{M\mathrm{PPM}}\left[n\right]$ is a binary sequence, the *M*PPM signal form (3) suggests that equalization schemes developed for ASK-QAM based transmission can be fruitfully employed when considering *M*PPM as well.

## 3. *M*PPM Receiver

**Figure 2:**
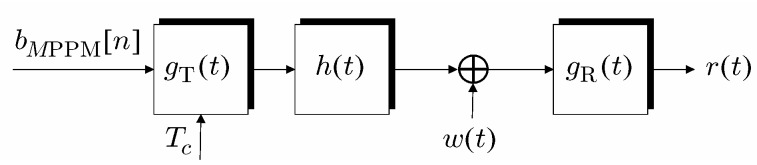
Chip based PPM transmission scheme.

As illustrated in Figure 2, after matched filtering at the receiver side we observe the signal:

(6) $$r\left(t\right)=\sum_{n=-\infty }^{+\infty }b_{M\mathrm{PPM}}\left[n\right]\;g\left(t-nT_{c}\right)+v\left(t\right).$$

In (6), $g\left(t\right)\;=\;\left(g_{{}_{T}}*h*g_{{}_{R}}\right)\left(t\right)$ denotes the overall impulse response that comprises also the matched filter $g_{{}_{R}}\left(t\right)\;=\;g_{{}_{T}}\left(-t\right)$ as well as the channel h(t), whereas $v\left(t\right)\;=\;\left(w*g_{{}_{R}}\right)\left(t\right)$ denotes independent additive noise w(t) observed after the matched filter, with * denoting the convolution operator.

The large bandwidth occupied by the *M*PPM signal (Figure 1) can be usefully exploited through fractional sampling of r(t) operating at rate $P/T_{c}$, where the fractional sampling integer factor *P* can assume values significantly greater than 1; for instance, the numerical results later presented in Section 5 have been obtained using P = 9.

**Figure 3:**
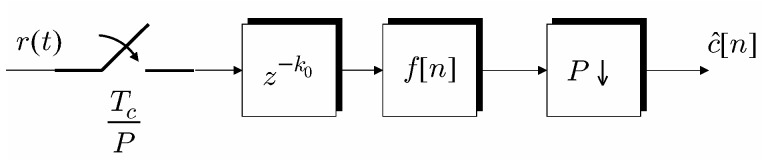
Chip based fractionally spaced equalization.

As illustrated in Figure 3 an FSE is a digital filter that operates on samples of r(t) taken at rate $P/T_{c}$, while yielding outputs at rate $1/T_{c}$; indicating with f[k] the impulse response of a finite impulse response (FIR) FSE of order *L*, the equalized sequence is:

(7) $$\hat{c}\left[n\right]=\sum_{k=0}^{L}f\left[k\right]\;r\left[nP-\left(k+k_{0}\right)\right].$$

In (7), $r\left[n\right]\;=\;{\left.r(t)\right|}_{t=nT_{c}/P}$ denotes the samples obtained by fractional sampling the received signal r(t), and $k_{0}\;\in \;N\left(P\right)\overset{\mathrm{def}}{=}\left\{0,1,\cdots ,P\;-\;1\right\}$ indicates the fractional sampling phase, the blind estimation of which is discussed in [17]. It is worth noting that the equalized sequence $\hat{c}\left[n\right]$ estimates the samples of the transmitted *M*PPM chips $b_{M\mathrm{PPM}}\left[n\right]$ only if the symbol timing has been already acquired, otherwise it simply furnishes an estimate of unsynchronized chips. Loosely speaking, the collection $\hat{c}\left[n\right]\overset{\mathrm{def}}{=}{\left[\hat{c}\left[n\right],\cdots ,\hat{c}\left[n\;-\;M\;+\;1\right]\right]}^{T}$ has one and only one non-zero sample if and only if symbol synchronization has been acquired; on the other hand, in the unsynchronized case it can also appear in collections $\hat{c}\left[n\right]$ that present two non-zero samples.

## 4. Trained and Blind Fractionally Spaced Equalization

Since (3) express the *M*PPM signal as a binary PAM signal, the determination of the FSE coefficients f[k] can be conducted in a blind fashion by a suitable modification of classical equalization techniques employed in ASK-QAM digital links. Nonetheless, significant performance improvement is expected when the peculiar spectral redundancy of *M*PPM signals is properly taken into account in the equalizer design. One of the aims of this contribution is to show how the minimum mean square error (MMSE) form of the so-called Bussgang blind equalization technique, firstly presented in [20] and then extended in [21,22,23,24,25], can be also applied to the *M*PPM signal representations (1), (2), and (3). Specifically, in the sequel the following three major items will be addressed:

1. The development of the MMSE nonlinearity that fully exploits the probabilistic description of the *M*PPM symbol formed as in (1);
2. The proof of how the probabilistic description of the *M*PPM symbol (1) can be employed to recover the symbol timing;
3. The introduction of a blind channel phase recovery technique that exploits the redundancy present in band-pass *M*PPM signals; it is worth highlighting that such a phase recovery stage is mandatory for band-pass transmission and coherent detection, and it is a critical step even in data aided (trained) equalization.

### 4.1. LMS Trained FSE

**Figure 4:**
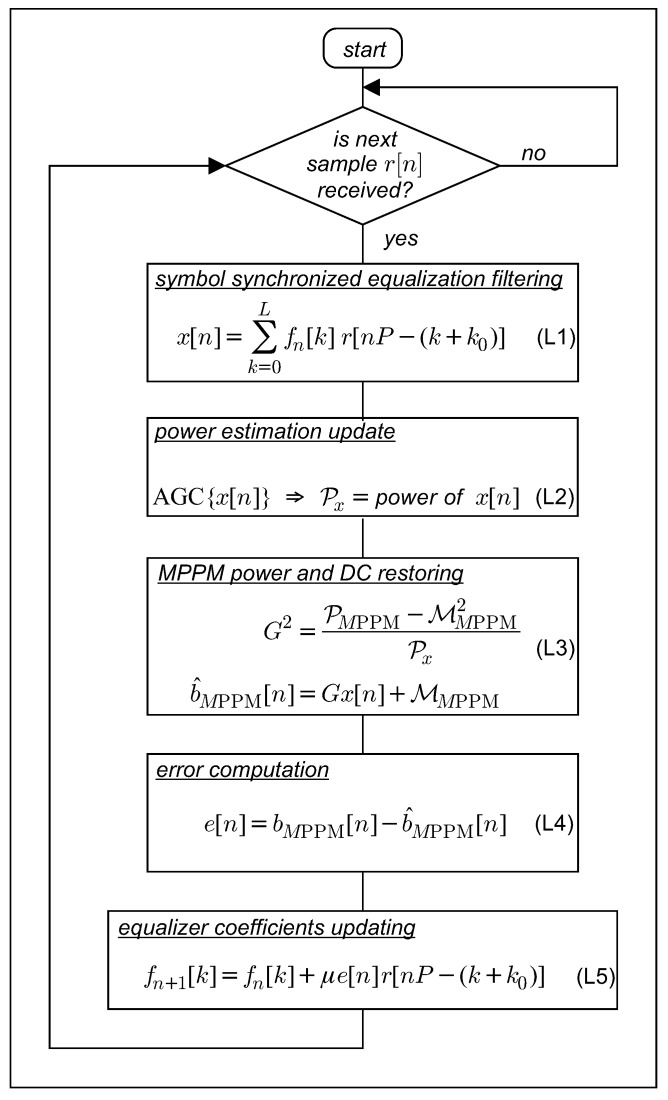
Least mean square (LMS) trained fractionally spaced equalizer (FSE) procedure. Direct component (DC).

The trained LMS implementation of a Bussgang type blind fractionally spaced equalization procedure, operated with the *learning factor* μ, is summarized in Figure 4.

### 4.2. LMS Blind Bussgang FSE

**Figure 5:**
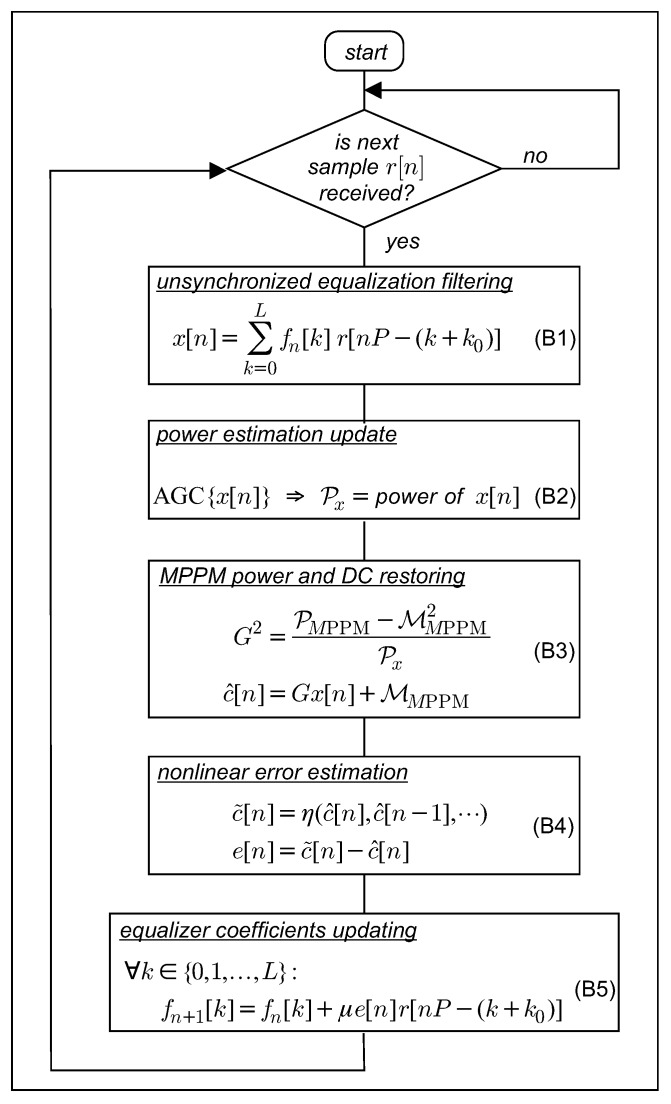
LMS blind Bussgang FSE procedure.

The LMS implementation of a Bussgang type blind fractionally spaced equalization procedure is summarized in Figure 5. In general, convergence of Bussgang equalizers is reached when $\hat{c}\left[n\right]$ becomes a Bussgang process satisfying the cross-correlation invariance $E\left\{\hat{c}\left[n\right]r\left[n\;-\;m\right]\right\}\;\propto \;E\left\{\tilde{c}\left[n\right]r\left[n\;-\;m\right]\right\}$ [20]; generalizations of Bussgang invariance and its application to image deblurring are found in [24,25,26,27].

*On the Nonlinear Error Estimation*: the *M* memory nonlinear *M*MNL transformation η(·) that obtains the error in (B4) is determined according to the MMSE criterion, the exploitation of which is well known to yield the MMSE estimator as the conditional a posteriori mean:

(8) $$\eta_{{}_{{}_{{}_{{}_{{}_{{}_{\mathrm{MMSE}}}}}}}}\;\left(c\left[n\right]\right)\;=\;E_{}\;\left\{c\left[n\right]|r\left[Pn\;-\;k_{0}\right],r\left[P\left(n\;-\;1\right)\;-\;k_{0}\right],\cdots \right\}.$$

It is worth noting that, since the chips c[n] are block correlated, the MMSE estimation (8) not only depends on the present received sample $r[Pn\;-\;k_{0}]$, but it also depends on all the past samples, namely $\left\{r\left[P\left(n\;-\;1\right)\;-\;k_{0}\right],r\left[P\left(n\;-\;2\right)\;-\;k_{0}\right],\cdots \right\}$.

Hence, as indicated in [23], to simplify the evaluation of (8) the following two assumptions are here retained:

A1. The expectation in (8) is conditionally taken with respect to the last *M* equalizer outputs:
  $$\hat{c}\left[n\right]\overset{\mathrm{def}}{=}{\left[\hat{c}\left[n\right],\cdots ,\hat{c}\left[n\;-\;M\;+\;1\right]\right]}^{T}$$
A2. The output of the FSE is assumed to satisfy the following additive white gaussian noise (AWGN) signal model:
  (9) $$\hat{c}\left[n\right]≃c\left[n\right]+w\left[n\right]$$
  being w[n] a realization of stationary white Gaussian random series of power $P_{{}_{\;w}}$ and statistically independent of $b_{M\mathrm{PPM}}\left[n\right]$.

It is worth noting that assumption A1 extends to the *M*PPM case already developed in [23] for correlated QAM binary symbols used in Global Systems for Mobile Communications digital links, and it is here applied to PPM for the first time.

The form assumed by the conditional a posteriori mean (8) when assumptions A1 and A2 are retained strictly depends on the set $S_{b}$ of all the possible transmitted binary *M*-tuples, indicated in the following by $c\left[n\right]\overset{\mathrm{def}}{=}{\left[c[n],\cdots ,c[n\;-\;M\;+\;1]\right]}^{T}$; this set can assume the following two different configurations, depending on whether symbol synchronization has been acquired or not:

1. *Sync*: $S_{c}\;=\;S_{c}^{\left(S\right)}$ collects all the binary *M*-tuples that have M − 1 zero valued elements, i.e., the *M* rows of the identity *M*-matrix $I_{M}$, so that its cardinality is *M*;
2. *Unsync*: $S_{c}\;=\;S_{c}^{\left(U\right)}$ joins $S_{c}^{\left(S\right)}$ with the binary *M*-tuple that has all zeros as well as with all the binary *M*-tuples that have M − 2 zeros; since these latter occurs in number of M(M − 1)/2, it results:
  $$\mathrm{card}\left(S_{c}^{\left(U\right)}\right)\;=\;1\;+\;M\;+\;\frac{M(M\;-\;1)}{2}\;=\;1+\frac{M(M\;+\;1)}{2}.$$

With $P\;\left\{c\right\}$ the probability of observing c, the signal model (9) leads to the following conditional *a posteriori* mean:

(10) $$\;E_{}\;\left\{c\left[n\right]\;\left|\hat{c}\left[n\right]\right.\right\}\;=\;\frac{\sum_{c\in S_{c}}P\;\left\{c\right\}\;c\exp\;\left(\frac{{∥\hat{c}\left[n\right]\;-\;c∥}_{2}^{2}}{2P_{{}_{\;w}}}\right)}{\sum_{c\in S_{c}}P\;\left\{c\right\}\exp\;\left(\frac{{∥\hat{c}\left[n\right]\;-\;c∥}_{2}^{2}}{2P_{{}_{\;w}}}\right).}$$

The evaluation of the probabilities $P\;\left\{c\right\}$ is reported in Appendix A. In more detail, in Appendix A we have analytically examined the two different cases of: (i) a single chip equal to 1 located at a generic position, and (ii) two chips equal to 1 distant *D* positions, respectively obtaining the expressions (A1) and (A2). Then, the numerical probability values for the most interesting cases of 2PPM, 4PPM and 8PPM are reported in Table 1, Table 2 and Table 3.

Finally, the MMNL $\eta (\hat{c}\left[n\right])$ in step 2 of Figure 5 selects the estimated chip corresponding to the first entry of the estimated vector $\tilde{c}\left[n\right]\;=\;E_{}\;\left\{c\left[n\right]\;\left|\hat{c}\left[n\right]\right.\right\}$ given in (10).

Once again, we outline that the MMNL estimator (10) exploits the block correlation nature of *M*PPM symbols and it is here presented for the first time.

When (10) acts on a single chip, i.e., $c\left[n\right]\;=\;\hat{c}\left[n\right]$ is formed using only the last equalized chip, the classical zero memory nonlinear (ZMNL) Bussgang procedure, as described in [21,22,23], is obtained.

## 5. Numerical Results

### 5.1. Severe Three-Paths Channel

**Figure 6:**
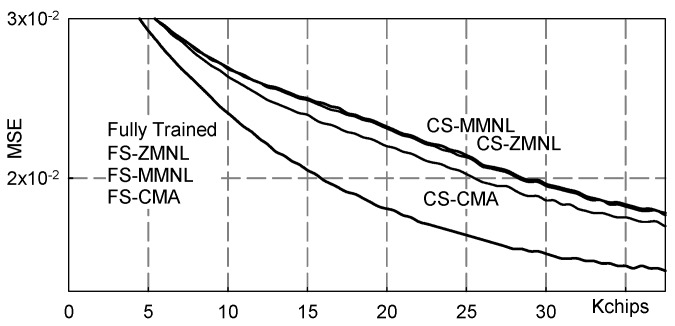
Mean square error (MSE) vs. transmitted chips: channel (11), NSR = 20dB.

**Figure 7:**
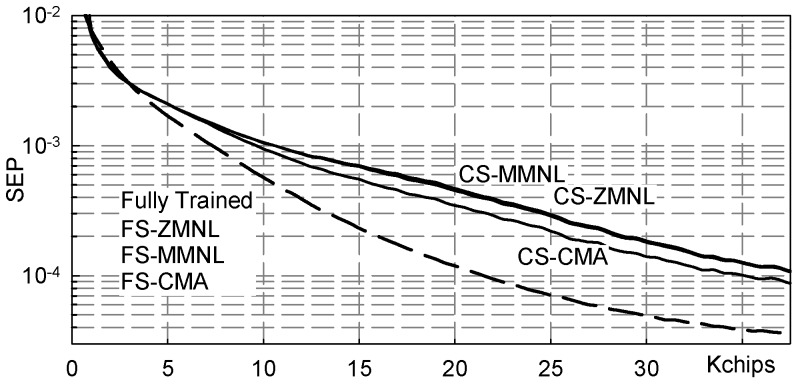
Symbol error probability (SEP) vs. transmitted chips: channel (11), NSR = 20dB.

Figure 6 and Figure 7 show numerical results referring to M = 2 and the following three-paths channel:

$$h_{3}\left(t\right)=\delta \left(t\right)-\delta \left(t-T_{c}\right)+\delta \left(t-2T_{c}\right).$$

Despite channel $h_{3}\left(t\right)$ having been discussed in [11] as a severely degrading one from the equalization point of view, we have considered the following, more challenging, scenario:

(11) $$h\left(t\right)=\left(h_{3}*w_{H,T_{c}}\right)\left(t\right)$$

with $w_{H,T_{c}}\left(t\right)$ being the Hamming window of duration $T_{c}$.

As in [11], we have considered a base-band transmission and, as far as the transmitting pulse is concerned, we have chosen a bell shaped one:

(12) $$g_{{}_{T}}\left(t\right)=A\exp\left(-t^{2}/2\alpha_{c}^{2}\right).$$

With the choice $\alpha \;=\;T_{c}/4$ the pulse duration is well approximated by $T_{c}$. Note that the scenario described in [11] is a pure discrete-time one, so the value of $T_{c}$ can be arbitrarily assumed since it only determines the frequency band in which the channel is used; when the real pulse (12) is employed, the channel bandwidth is approximately $1/T_{c}$. Moreover, the amplitude *A* has been chosen to be equal and determined so to have unit energy. Lastly, the fractional sampling factor has been chosen as P = 9, i.e., the minimum one that still allows to full exploitation of the spectral redundancy offered by the *M*PPM signal.

In more detail, we have numerically evaluated the mean square error (MSE) measured at the equalizer output defined as follows:

MSE=ISI+ONP

where ISI and the overall noise power (ONP) contributions are defined as follows:

(13) $$\begin{array}{cc} \mathrm{ISI} & =E_{o_{s}}-1 \end{array}$$

(14) $$\begin{array}{cc} \mathrm{ONP} & =\mathrm{NSR}\times E_{o_{w}}. \end{array}$$

In (13), $E_{o_{s}}$ denotes the energy of the overall channel/equalizer impulse response seen by the useful signal:

(15) $$o_{s}\left[n\right]=\left(g*f\right)\left[\left(n-k_{0}\right)P\right].$$

In (14), NSR denotes the noise-to-signal ratio and $E_{o_{w}}$ the energy of the overall channel/equalizer impulse response seen by the noise:

(16) $$o_{w}\left[n\right]=\left(g_{{}_{R}}*f\right)\left[\left(n-k_{0}\right)P\right].$$

In Figure 6 results pertaining to the following equalizers are reported:

- Fully Trained: the ideal data-aided fractionally spaced equalizer that knows all the transmitted symbols;
- FS-MMNL: the blind fractionally spaced Bussgang equalizer that uses the novel *M*-memory nonlinearity (B4) here presented;
- FS-ZMNL: the blind fractionally spaced Bussgang equalizer that uses the zero-memory nonlinearity described in [23];
- FS-CMA: the blind fractionally spaced CMA equalizer [28];
- CS-MMNL: the blind chip spaced Bussgang equalizer that uses the novel *M*-memory nonlinearity (B4);
- CS-ZMNL: the blind chip spaced Bussgang equalizer that uses the zero-memory nonlinearity described in [23];
- CS-CMA: the blind chip spaced CMA equalizer [28].

It is worth noting that chip-spaced equalization is obtained by setting P = 1 in any fractionally spaced equalization scheme.

The values reported in Figure 6 have been obtained by averaging over 100 independent Monte Carlo runs and plotted as equalized chips go by; it is evident that FSE achieves a significant performance improvement with respect to chip spaced equalization. Moreover, it is also appreciated how both MMNL and ZMNL blind equalization schemes approach the performance achieved by the “fully trained” equalizer, i.e., the ideal data aided adaptive equalizer that knows all the transmitted chips. For comparison purposes, we have also reported the accuracy achieved by the blind constant modulus algorithm (CMA) [28].

In order to give an idea about the achievable symbol error rate, Figure 7 reports the symbol error probability (SEP) calculated considering the MSE values of Figure 6 as they were the power of additive white Gaussian noise:

(17) $$\mathrm{SEP}=\frac{1}{2}\mathrm{erfc}\left(\frac{1}{2\sqrt{2\mathrm{MSE}}}\right).$$

### 5.2. Multipath Channel

The numerical results reported below still refer to the M = 2 case, but the channel is a typical underwater multipath one [3]:

(18) $$h_{Z}\left(t\right)=\sum_{k=0}^{7}A_{k}\;\delta \left(t-\tau_{k}\right).$$

The parameters of the multipath channel (18) are found in Table 4.

As already done in [3], to have the channel (18) used in the bandwidth of 8kHz centered around $f_{0}\;=\;4\;\mathrm{kHz}$, the simulation has been operated with the following, envelope bell shaped, transmitting pulse:

(19) $$\begin{array}{cc} g_{{}_{T}}\left(t\right) & =A_{c}\exp\left(-t^{2}/2\alpha_{c}^{2}\right)\cos\left(2\pi f_{0}t\right)-A_{s}\exp\left(-t^{2}/2\alpha_{s}^{2}\right)\sin\left(2\pi f_{0}t\right). \end{array}$$

Details on complex low-pass representation and filtering of band-pass *M*PPM signals are given in Appendix B.

With the choice of $\alpha_{c}\;=\;\alpha_{s}\;=\;T_{c}/4$, the pulse duration is well approximated by $T_{c}$ and the pulse bandwidth is $B\;≃\;8/T_{c}$, so that $T_{c}\;=\;1\mathrm{ms}$ yields just B ≃ 8kHz; moreover, the amplitudes $A_{c}$ and $A_{s}$ have been chosen equal and determined so as to have unit energy, and the fractional sampling factor has been chosen P = 9.

**Figure 8:**
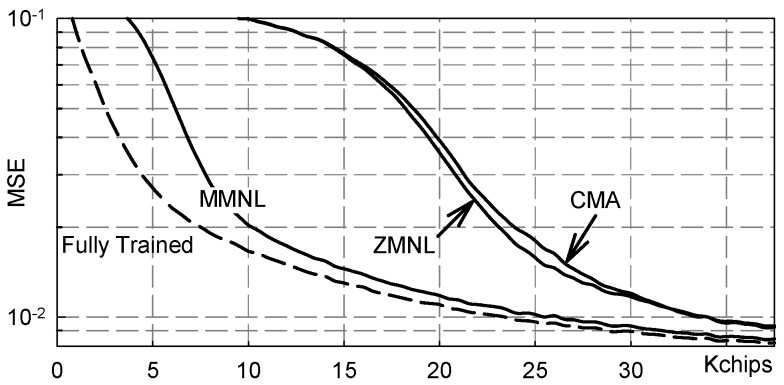
MSE vs. transmitted chips: channel (20), NSR = 20dB.

Figure 8 shows numerical results referring to the following, more challenging from the equalization point of view, channel:

(20) $$h\left(t\right)=\left(h_{Z}*w_{H,D_{1}}\right)\left(t\right)$$

where $w_{H,D_{1}}\left(t\right)$ is the Hamming window of duration $D_{1}\;=\;\bar{4.3}\;\mathrm{ms}$.

The values reported in Figure 8 have been obtained by averaging over 100 independent Monte Carlo runs and it is clear that there is significant performance improvement achieved by the novel MMNL based blind equalization scheme with respect to both the CMA and ZMNL based ones. Moreover, we can also appreciated how the MMNL equalizer performance is very close to the ideal “fully trained” equalizer performance.

**Figure 9:**
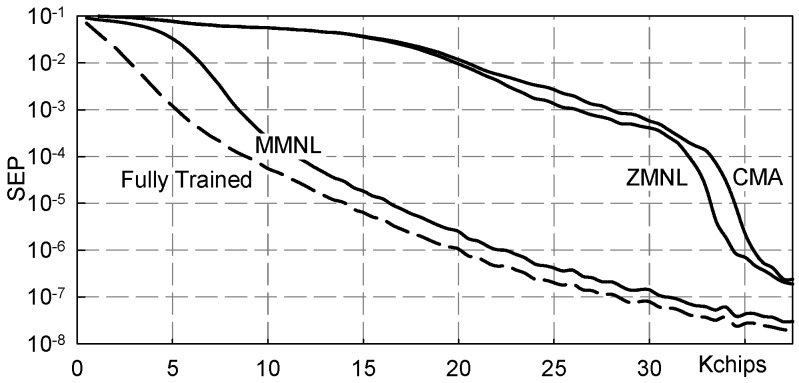
SEP vs. transmitted chips: channel (20), NSR = 20dB.

As above, to give an idea about the achievable error rate, Figure 9 reports the symbol error probability (SEP) calculated using (17).

*Sampling Phase Sensitivity*: The results shown in Figure 8 and Figure 9 have been obtained by averaging on three different sampling phase values, namely $k_{0}\;=\;0,1,6$ as these latter result from the fourth order statistic based sampling phase estimation technique presented in [17]. While the MMNL based blind equalization scheme has reached convergence in all the Monte Carlo runs, in the CMA based blind equalization scheme, a significant convergence failure percentage has been observed, on average equal to about 7.5%. Hence, and quite interestingly, the novel MMNL based blind equalization scheme guarantees superior performance also in terms of sampling phase sensitivity.

### 5.3. Severe Multipath Channel

**Figure 10:**
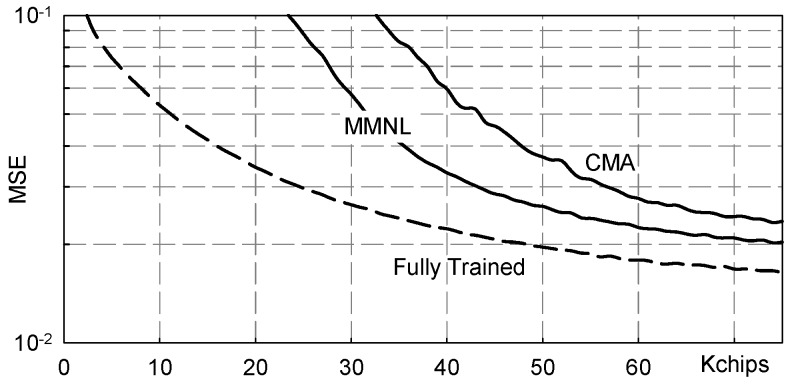
MSE vs. transmitted chips: channel (21), NSR = 20dB.

**Figure 11:**
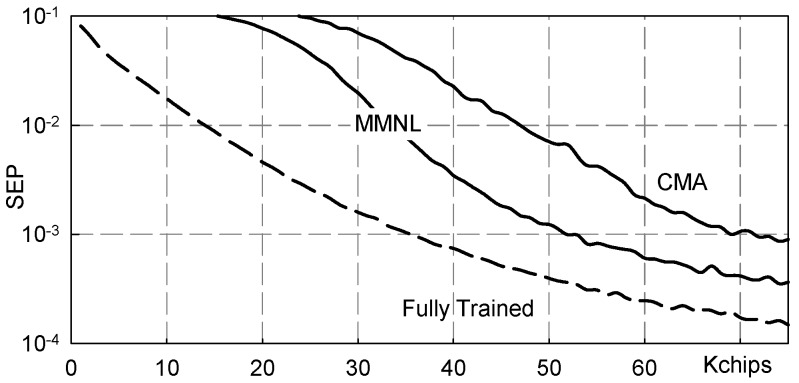
SEP vs. transmitted chips: channel (21), NSR = 20dB.

Figure 10 and Figure 11 show numerical results referring to the M = 4 case and to the following severe multipath channel:

(21) $$h\left(t\right)=\left(h_{Z}*w_{H,D_{2}}\right)\left(t\right)$$

where $w_{H,D_{2}}\left(t\right)$ is the Hamming window of duration $D_{2}\;=\;\bar{8.3}\;\mathrm{ms}$.

In this case, the ZMNL based blind equalization scheme has never reached convergence, therefore no numerical results have been obtained; on the other hand, the MMNL based blind equalization scheme confirms its superior performance with respect to the CMA based one.

*Sampling Phase Sensitivity*: Figure 10 and Figure 11 show the FSE performance reporting the results obtained by averaging on three sampling phases, namely $k_{0}\;=\;0,7,8$, selected according to the fourth order statistic based sampling phase estimation technique already employed when considering the scenario introduced in the previous Subsection *B*. The trend of the plotted curves show how the MMNL based blind equalization scheme outperforms the CMA based blind equalization scheme in terms of both SEP and convergence speed. Furthermore, while the MMNL scheme has reached convergence in all the Monte Carlo runs, a significant misconvergence percentage has been observed, on the average equal to about 10%, for the CMA scheme. Therefore, even dealing with this more severe scenario, the novel MMNL based blind equalization scheme offers lower sampling phase sensitivity, making its performance the best one.

## 6. Conclusions

Using a chip based representation, we have been able to exploit the memory of PPM signals through fractional sampling operated at the receiver side, cascaded with digital filtering aimed at restoring the channel equalization condition. The success of this approach is mainly due to the spectral redundancy offered by *M*PPM signals. The design of the fractionally spaced equalizer has been conducted in a blind, not data aided, fashion and the resulting novel technique has been analyzed and its performance assessed by numerical simulation. Numerical results have confirmed that the here presented novel blind equalization technique offers better performance with respect to the classical CMA blind equalization algorithms, both in terms of accuracy as well as sampling phase sensitivity.

These results show the feasibility of the PPM single carrier approach in severe multipath channel scenarios where, in principle, OFDM based transmission may be failing. Since the here presented PPM FSE is blind, performance comparison with data-aided OFDM is unfair. In this regard, further studies will be devoted to devise a trained PPM FSE, the performance of which can be fairly discussed and compared to those of OFDM based schemes operating in more specifically defined severe multipath channel scenarios.

## Figures and Tables

**Table 1:** Probabilites of M = 2 asynchronous chips.

*p*	*D*	*M* = 2 Chips Combinations	Probability
-	-	00	1/8
0	-	10	3/8
1	-	01	3/8
0	1	11	1/8

**Table 2:** Probabilites of M = 4 asynchronous chips.

*p*	*D*	*M* = 4 Chips Combinations	Probability
-	-	0000	10/64
0	-	1000	10/64
1	-	0100	12/64
2	-	0010	12/64
3	-	0001	10/64
0	1	1100	1/64
	2	1010	2/64
	3	1001	3/64
1	1	0110	1/64
	2	0101	2/64
2	1	0011	1/64

**Table 3:** Probabilites of M = 8 asynchronous chips.

*p*	*D*	*M* = 8 Chips Combinations	Probability
-	-	00000000	84/512
0	-	10000000	36/512
1	-	01000000	42/512
2	-	00100000	46/512
3	-	00010000	48/512
4	-	00001000	48/512
5	-	00000100	46/512
6	-	00000010	42/512
7	-	00000001	36/512
0	1	11000000	1/512
	2	10100000	2/512
	3	10010000	3/512
	4	10001000	4/512
	5	10000100	5/512
	6	10000010	6/512
	7	10000001	7/512
1	1	01100000	1/512
	2	01010000	2/512
	3	01001000	3/512
	4	01000100	4/512
	5	01000010	5/512
	6	01000001	6/512
2	1	00110000	1/512
	2	00101000	2/512
	3	00100100	3/512
	4	00100010	4/512
	5	00100001	5/512
3	1	00011000	1/512
	2	00010100	2/512
	3	00010010	3/512
	4	00010001	4/512
4	1	00001100	1/512
	2	00001010	2/512
	3	00001001	3/512
5	1	00000110	1/512
	2	00000101	2/512
6	1	00000011	1/512

**Table 4:** Multipath channel parameters.

ray number	*k*	>0	1	2	3	4	5	6	7
ray amplitude	$A_{k}$	0.808	1.0	0.796	0.461	0.522	0.831	0.421	0.725
ray delay	$\tau_{k}$ (ms)	0	18.6	30.0	59.3	61.0	62.9	91.3	107.9

**Figure A1:**
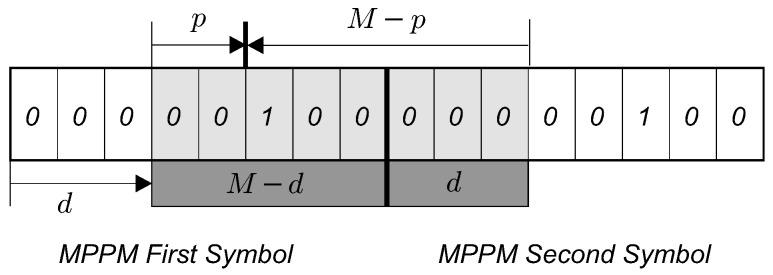
Single chip 1 at position *p*.

**Figure A2:**
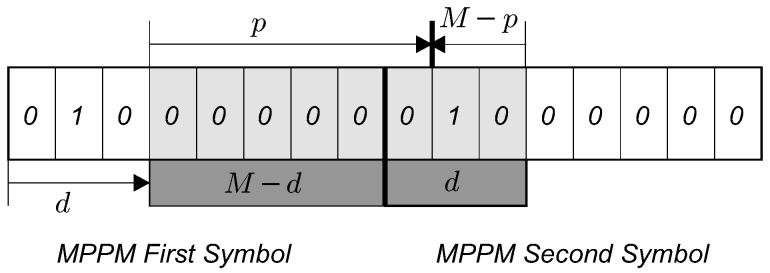
Single chip 1 at position *p*.

**Figure A3:**
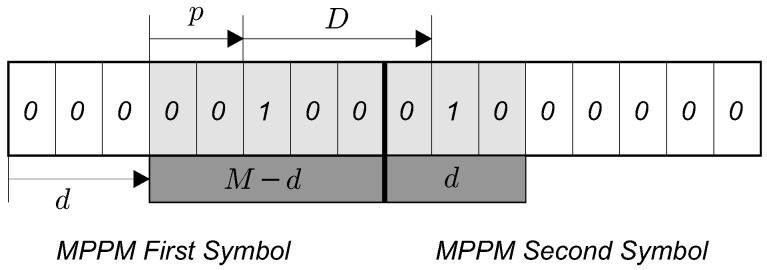
Two chips 1 distant *D* positions.

**Figure A4:**
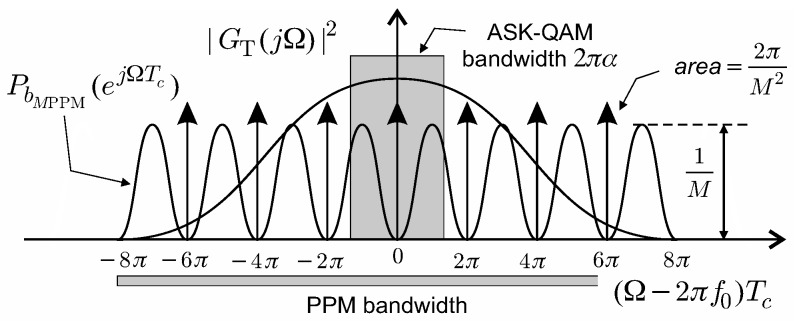
Spectral representation of band-pass PPM signals.

**Figure A5:**
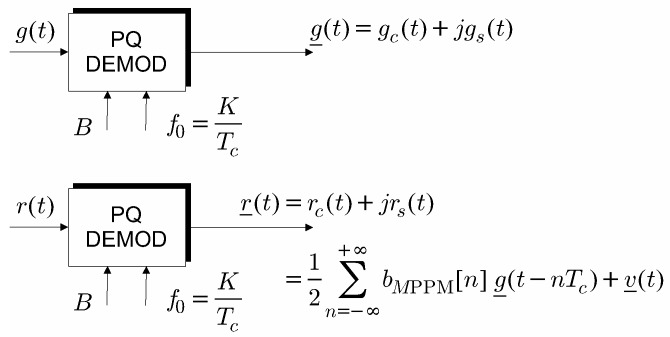
Low-pass signal representation of *M*PPM signals.

**Figure A6:**
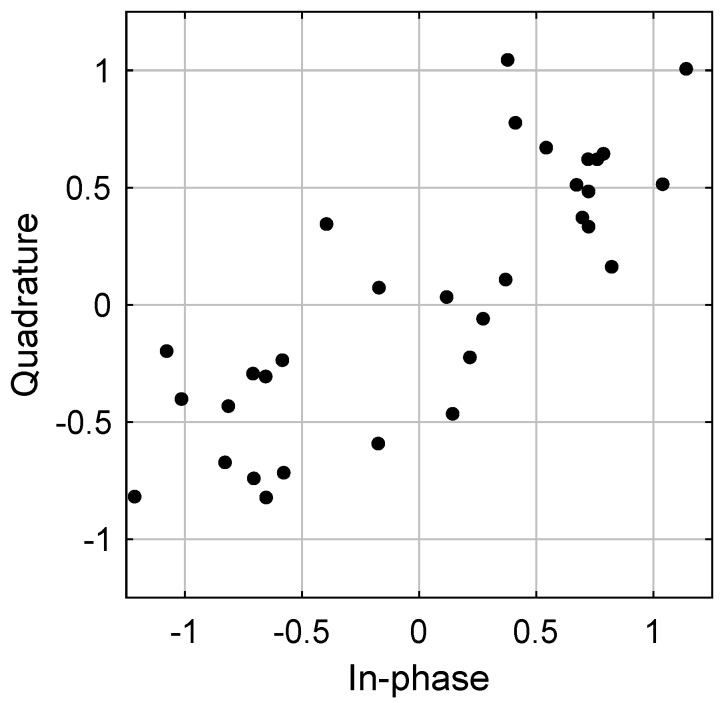
Snapshot of samples of the complex signal $\underline{r}\left(nT_{c}\right)$.

**Figure A7:**
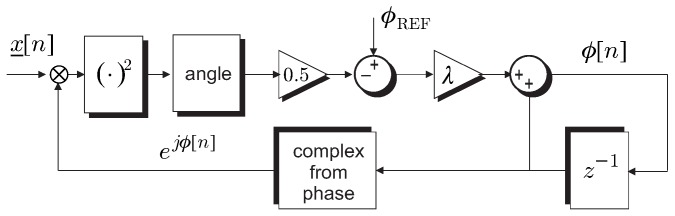
Block diagram of automatic phase control loop.

## Appendix A. Binary M-Tuple Probabilities Evaluation

### Appendix A.1. Case 1: Single Chip 1 at Position p

For each delay *d*, we have M − d chips belonging to the first *M*PPM symbol and *d* chips belonging to the second *M*PPM symbol, which is statistically independent of the first one. The number of possible cases are *M*, see Table 1, Table 2 and Table 3, but the following two different situations can occur, see Figure A1 and Figure A2 with 0 ≤ p ≤ M − 1:

d < M − p: in this case, see Figure A1, the single chip having value equal to 1 at position *p* belongs to the first *M*PPM symbol; hence, the first M − d chips occur with probability 1/M, while the second *d* chips are all 0 and occur with probability (M − d)/M.

d ≥ M − p: in this case, see Figure A2, the single chip having value equal to 1 at position *p* belongs to the second *M*PPM symbol; hence, the second *d* chips occur with probability 1/M, while the first M − d chips are all 0 and occur with probability d/M.

The total probability is obtained through averaging over all the equiprobable delays d = 0,1,⋯M − 1:

(A1) $$\begin{array}{cc} P\;\left\{\mathrm{single}1\mathrm{at}p\right\} & =\frac{1}{M}\left[\sum_{d=0}^{M-1-p}\;\frac{1}{M}\frac{M\;-\;d}{M}\right.+\left.\;\sum_{d=M-p}^{M-1}\;\frac{1}{M}\frac{d}{M}\right]\\ & =\frac{1}{M^{3}}\left[M\left(M\;-\;p\right)-\frac{\left(M\;-\;p)(M\;-\;1\;-\;p\right)}{2}\right.\left.+\frac{M(M\;-\;1)}{2}-\frac{\left(M\;-\;p)(M\;-\;1\;-\;p\right)}{2}\right]\\ & =\;\frac{1}{M^{3}}\left[\frac{M^{2}}{2}+\frac{M}{2}+pM\;-\;p\;-\;p^{2}\right]\;=\;\frac{1}{M^{3}}\left[\frac{M(M+1)}{2}+p\left(M\;-\;1\;-\;p\right)\right] \end{array}$$

As expected $P\;\left\{\mathrm{single}1\mathrm{at}p\right\}$ $=\;P\;\left\{\mathrm{single}1\mathrm{at}M-1-p\right\}$.

### Appendix A.2. Case 2: Two Chips 1 Distant D Positions

In this situation, see Figure A3 with 0 ≤ p ≤ M − 1 and 1 ≤ D ≤ M − 1, one chip 1 belongs to the first *M*PPM symbol while the other chip 1 belongs to the second *M*PPM symbol, which is statistically independent of the first one. The number of possible cases is M(M − 1)/2, see Table 1, Table 2 and Table 3, and the first M − d chips always occur with probability 1/M but also the second *d* chips always occur with the same probability 1/M.

It is worth noting that both those probabilities does not depend on the delay *d*, but only *D* delays are compatible with the distance *D*; in fact, said *p* the position of the first chip 1, from Figure A3 we see that the first chip 1 belongs to the first *M*PPM symbol iff d + p ≤ M − 1, while the second chip1 belongs to the second *M*PPM symbol iff d + D + p ≥ M − 1, so that the compatible delays are d = M − p − D,⋯,M − p − 1, and their number is *D*.

The total probability is obtained through averaging over all the *D* compatible delays:

(A2) $$\begin{array}{cc} P\;\left\{1\mathrm{at}p\Lambda 1\mathrm{at}p\;+\;D\right\} & \;=\;\frac{1}{M}\sum_{d=M\;-\;p-D}^{M\;-\;p\;-\;1}\frac{1}{M^{2}}\;=\;\frac{D}{M^{3}} \end{array}$$

## Appendix B. Complex Low-Pass Representation of Band-Pass MPPM Signals

The complex low-pass representation of band-pass signals stems from the following signal form:

(A3) $$\begin{array}{cc} g(t) & =g_{c}\left(t\right)\cos\left(2\pi f_{0}t\right)-g_{s}\left(t\right)\sin\left(2\pi f_{0}t\right)\\ & =ℜ\left\{\left(g_{c}\left(t\right)+jg_{s}\left(t\right)\right)\;e^{j2\pi f_{0}t}\right\} \end{array}$$

The representation (A3) is unique when the complex low-pass signal $\underline{g}\left(t\right)\;=\;g_{c}\left(t\right)+jg_{s}\left(t\right)$ is bandlimited with bandwidth $B\;<\;2f_{0}$. The extraction of the low-pass components $g_{c}\left(t\right)$ and $g_{s}\left(t\right)$ is achieved by classical phase-quadrature demodulation, graphically indicated as in Figure A5.

Without loss of generality, let us now consider the case $f_{0}\;=\;k/T_{c}$ with $k\;\in \;N^{+}$, in which the spectra of Figure 1 show up as in Figure A4.

Bearing in mind (6), it is simple matter to verify the equivalence shown in Figure A5.

In other words, even though the physical mechanism is different, *M*PPM signals admit a complex low-pass representation analog to QAM signals; this in turns means that complex equalization filtering can be realized after matched filtering and phase-quadrature demodulation operated at receiver side.

### Appendix Automatic Phase Controls

Since $b_{M\mathrm{PPM}}\left[n\right]$ is a binary sequence, the sampled complex low-pass received signal $\underline{r}\left(nT_{c}\right)$ (6) results improper complex [29]. As shown in Figure A6, this in turn implies that, due to both noise and channel effects, the complex samples $\underline{r}\left(nT_{c}\right)$ result to be scattered around a straight line whose orientation is substantially determined by the channel phase.

In summary, the improper complexity of $\underline{r}\left(nT_{c}\right)$ can be fruitfully exploited to devise blind estimation of the phase rotation induced by the channel. Since the application of the nonlinearity (16) requires such a phase to be already compensated, we have experienced good results using the following simple automatic phase control loop, to be operated with $\phi_{{}_{\mathrm{REF}}}\;=\;0$ and also sketched in Figure A7.

(A4) $$\;\phi \left[n\right]=\phi \left[n\;-\;1\right]\;+\;\lambda \left[\phi_{{}_{\mathrm{REF}}}-\frac{1}{2}\mathrm{angle}\left(\;x\left[n\right]\;e^{j\phi [n\;-\;1]}\right)\;\right]$$

Since the phase loop (A4) retrieves the phase modulo π, it remains a sign ambiguity to resolve by using differential encoding or exploiting the skewness of *M*PPM signals for M ≥ 4.

## References

[1] Stojanovic M., Preisig J. (2009). Underwater acoustic communication channels: Propagation models and statistical characterization. IEEE Commun. Mag..

[2] Proakis J.G. (1991). Adaptive equalization techniques for acoustic telemetry channels. IEEE J. Ocean. Eng..

[3] Zhao S., Zhang X., Zhang X. Iterative frequency domain equalization combined with LDPC decoding for single-carrier underwater acoustic communications. Proceedings of the OCEANS 2016 MTS/IEEE Monterey.

[4] Liu L., Zhang Y., Zhang P., Zhou L., Niu J. Channel coding for underwater acoustic single-carrier CDMA communication system. Proceedings of the 7th International Conference on Electronics and Information Engineering.

[5] Chithra K., Sireesha N., Thangavel C., Gowthaman V., Narayanan S.S., Sudhakar T., Atmanand M.A. (2015). Underwater communication implementation with OFDM. Indian J. Geo-Mar. Sci..

[6] Hessien S., Tokgöz S.C., Anous N., Boyacı A., Abdallah M., Qaraqe K.A. (2018). Experimental evaluation of OFDM-based underwater visible light communication cystem. IEEE Photonics J..

[7] Ashri R., Shaban H., El-Nasr M. (2017). A novel fractional Fourier transform-based ASK-OFDM system for underwater acoustic communications. Appl. Sci..

[8] Liu S., Ma T., Qiao G., Ma L., Yin Y. (2017). Biologically inspired covert underwater acoustic communication by mimicking dolphin whistles. Appl. Acoust..

[9] Li B., Huang J., Zhou S., Ball K., Stojanovic M., Freitag L., Willett P. (2009). MIMO-OFDM for high-rate underwater acoustic communications. IEEE J. Ocean. Eng..

[10] Stojanovic M. OFDM for underwater acoustic communications: Adaptive synchronization and sparse channel estimation. Proceedings of the 2008 IEEE International Conference on Acoustics, Speech and Signal Processing.

[11] Klein A.G., Johnson C.R. MMSE decision feedback equalization of pulse position modulated signals. Proceedings of the EEE International Conference on Communications.

[12] Ungerboeck G. (1976). Fractional tap-spacing equalizer and consequences for clock recovery in data modems. IEEE Trans. Commun..

[13] Scarano G., Petroni A., Biagi M., Cusani R. (2017). Second-order statistics driven LMS blind fractionally spaced channel equalization. IEEE Signal Process. Lett..

[14] Xiao Y. (2013). Recursive least squares fractionally-spaced blind equalization algorithm for underwater acoustic communication. J. Inf. Comput. Sci..

[15] Artman D.J., Chari S., Gooch R.P. Joint equalization and timing recovery in a fractionally-spaced equalizer. Proceedings of the 1992 Conference Record of the Twenty-Sixth Asilomar Conference on Signals, Systems and Computers.

[16] Nasir A.A., Durrani S., Kennedy R.A. Blind fractionally spaced equalization and timing synchronization in wireless fading channels. Proceedings of the 2010 2nd International Conference on Future Computer and Communication.

[17] Scarano G., Petroni A., Cusani R., Biagi M. Sampling phase estimation in underwater PPM fractionally sampled equalization. Proceedings of the 26th European Signal Processing Conference (EUSIPCO).

[18] Biglieri E., Benedetto S. (1999). Principles of Digital Transmission.

[19] Scarano G. Lezioni di Elaborazione Statistica dei Segnali (Vol.II).

[20] Godfrey R., Rocca F. (1981). Zero memory non-linear deconvolution. Geophys. Pros..

[21] Bellini S., Haykin S.S. (1994). Bussgang Techniques for Blind Deconvolution and Equalization. Blind Deconvolution.

[22] Jacovitti G., Panci G., Scarano G. (2001). Bussgang-zero crossing equalization: An integrated HOS-SOS approach. IEEE Trans. Signal Process..

[23] Panci G., Colonnese S., Campisi P., Scarano G. (2005). Fractionally spaced Bussgang equalization for correlated input symbols: A Bussgang approach. IEEE Trans. Signal Process..

[24] Panci G., Colonnese S., Campisi P., Scarano G. (2003). Multichannel blind image deconvolution using the Bussgang algorithm: Spatial and multiresolution approaches. IEEE Trans. Image Process..

[25] Colonnese S., Campisi P., Panci G., Scarano G. (2004). Blind image deblurring driver by nonlinear processing in the edge domain. EURASIP J. Appl. Signal Process..

[26] Scarano G. (1991). Cumulant series expansion of hybrid nonlinear moment of complex random variables. IEEE Trans. Signal Process..

[27] Scarano G., Caggiati D., Jacovitti G. (1993). Cumulant series expansion of hybrid nonlinear moments of n variates. IEEE Trans. Signal Process..

[28] Godard D.N. (1980). Self-recovering equalization and carrier tracking in two-dimensional data communications systems. IEEE Trans. Commun..

[29] Schreier P.J., Scharf L.L. (2010). Statistical Signal Processing of Complex-Valued Data: The Theory of Improper and Noncircular Signals.

